# Trace biomarkers associated with spontaneous preterm birth from the maternal serum metabolome of asymptomatic nulliparous women – parallel case-control studies from the SCOPE cohort

**DOI:** 10.1038/s41598-019-50252-7

**Published:** 2019-09-23

**Authors:** Renato T. Souza, Elizabeth J. McKenzie, Beatrix Jones, Jamie V. de Seymour, Melinda M. Thomas, Erica Zarate, Ting Li Han, Lesley McCowan, Karolina Sulek, Silas Villas-Boas, Louise C. Kenny, José G. Cecatti, Philip N. Baker

**Affiliations:** 1 Department of Obstetrics and Gynecology, University of Campinas, Campinas, Brazil; 2 The University of Auckland, Auckland, New Zealand; 3 The Department of Women’s and Children’s Health, Institute of Translational Medicine, Faculty of Health and Life Sciences, University of Liverpool, Liverpool, UK; 4 College of Life Sciences, University of Leicester, Leicester, United Kingdom

**Keywords:** Predictive markers, Predictive markers

## Abstract

Prediction of spontaneous preterm birth (sPTB) in asymptomatic women remains a great challenge; accurate and reproducible screening tools are still not available in clinical practice. We aimed to investigate whether the maternal serum metabolome together with clinical factors could be used to identify asymptomatic women at risk of sPTB. We conducted two case-control studies using gas chromatography-mass spectrometry to analyse maternal serum samples collected at 15- and 20-weeks’ gestation from 164 nulliparous women from Cork, and 157 from Auckland. Smoking and vaginal bleeding before 15 weeks were the only significant clinical predictors of sPTB for Auckland and Cork subsets, respectively. Decane, undecane, and dodecane were significantly associated with sPTB (FDR < 0.05) in the Cork subset. An odds ratio of 1.9 was associated with a one standard deviation increase in log (undecane) in a multiple logistic regression which also included vaginal bleeding as a predictor. In summary, elevated serum levels of the alkanes decane, undecane, and dodecane were associated with sPTB in asymptomatic nulliparous women from Cork, but not in the Auckland cohort. The association is not strong enough to be a useful clinical predictor, but suggests that further investigation of the association between oxidative stress processes and sPTB risk is warranted.

## Introduction

Spontaneous preterm birth (sPTB) due to spontaneous onset of labour or premature rupture of membranes (PROM) is a major cause of neonatal mortality and morbidity^1–3^. Although sPTB is prevalent in both high and low/middle-income countries, the major burden of sPTB is concentrated in Asian and African countries where about 85% of preterm births occur^1,4,5^. Short- and long-term consequences of preterm birth include bronchopulmonary dysplasia, neurodevelopment and cognitive impairment, retinopathy, as well as substantial impact on the functional, mental and social health of the infant and its family^6–8^. Despite an increase in the research conducted into sPTB and advancements in the implementation of management and prevention strategies, significant reductions of sPTB have not been achieved^9,10^.

Natural progesterone (oral or vaginal), 17α-hydroxyprogesterone (intra-muscular), pessary and/or cervical cerclage have been variously recommended to prevent sPTB in some guidelines^11–14^, but their benefits are disputed. According to systematic reviews and meta-analyses, the use of such interventions in selected women can reduce the risk for sPTB, but they are less likely to reduce the risk for perinatal morbidity^15–19^. The identification of a more accurate screening tool to predict which women are most at risk of sPTB could improve the selection of women that would benefit from interventions, increasing the likelihood of success and reducing the costs to the healthcare system. However, current screening tools used to identify women at risk of developing sPTB fail to accurately predict preterm birth in asymptomatic women.

Cervical length is possibly the most employed indicator for sPTB risk in clinical practice. While there is evidence for an increased risk of sPTB in women with a shortened cervix^11,12^, approximately 2/3 of women with short cervical length will have a term birth^20,21^. Chemical biomarkers for sPTB have also been sought, with fetal fibronectin (fFN), insulin-like growth factor-binding protein 1 (IGFPB-1), and interleukin-6 (IL-6)^22–27^ among the most studied. A systematic review of sPTB biomarkers found more than 200 studies published between 1965 and 2008, reporting more than 100 potential biomarkers^27^. However, no single biomarker has proven to be a reliable predictor of sPTB. The authors of the systematic review concluded that there are many heterogeneities between the studies in terms of their experimental study design, timepoint of sample collection, and sample processing methods. One of the largest prospective cohort studies to date evaluated the performance of serial transvaginal cervical length measurements and quantitative vaginal fFN levels for predicting sPTB in a sample of approximately 10,000 nulliparous women with singleton pregnancies^28^. Despite determination of cervical length and fibronectin levels at three different gestations, the model still showed low predictive accuracy for sPTB.

sPTB is a condition with a multifactorial aetiology, a long pre-clinical phase, and adaptive mechanisms during pregnancy^29^. For instance, the cervical remodelling process, which involves softening and ripening of the cervix, invariably occurs in the spontaneous onset of labour and is considered its endpoint^30^. However, it does not occur in the same period of pregnancy for all women, which limits its potential as a marker for the prediction of sPTB^31^. The underlying complexity of the drivers of sPTB demands a robust analytical technique capable of taking into consideration the multiple pathways potentially affected in the development of sPTB.

Metabolomics, the study of low molecular weight compounds in a biological system, has previously been used to successfully investigate pregnancy complications such as fetal growth restriction, preeclampsia, and gestational diabetes mellitus^32–35^. Metabolomic measurements provide a biochemical snapshot of the physiological state of the organism^36^; their close relationship with the biological phenotype means they have the potential to reveal underlying mechanisms of disease. Due to the biochemical complexity of the metabolome, hyphenated analytical techniques are used. This involves subjecting sample extracts to a high-resolution separation technique such as gas chromatography (GC), high-pressure liquid chromatography (HPLC), or capillary electrophoresis (CE). Identification is then carried out using mass spectrometry (MS), or nuclear magnetic resonance (NMR).

The Preterm SAMBA study (Preterm Screening and Metabolomics in Brazil and Auckland) has been established to identify and validate metabolomic biomarkers for sPTB^37^, in collaboration with the SCOPE (Screening for Pregnancy Endpoints) study. SCOPE was an international multicentre prospective cohort study that collected standardized data and samples from more than 5,690 low-risk pregnant women^38–40^. The main objective of SCOPE was to develop a robust database and biobank to identify predictors and develop screening tests for preeclampsia, sPTB and small-for-gestational-age newborns. The current study describes the results of Preterm SAMBA phase 1, which used the SCOPE biobank to investigate the metabolomic profile, at 15- and 20-weeks’ gestation, amongst SCOPE participants who went on to have sPTB, when compared to matched controls. Parallel analyses were performed using participants from the Irish and New Zealand’s centres of the SCOPE study.

## Methods

### Study design and participants

Two case-control studies were conducted, using a sub-set of women from the Auckland, New Zealand and Cork, Ireland centres of the Screening for Pregnancy Endpoints (SCOPE) study^41^.

The SCOPE study recruited 5,690 nulliparous low-risk women with a singleton pregnancy from New Zealand, Australia, Ireland and the United Kingdom between November 2004 and August 2008. Ethical approval was obtained from the local ethics committees in both Ireland and New Zealand (Cork: study number ECM5 (10) 05/02/08, Clinical Research Ethics Committee of the Cork Teaching Hospitals; New Zealand: study number AKX/02/00/364, Northern X Reginal Ethics Committee) and all participants gave their written informed consent. Details on enrolment and inclusion criteria for the SCOPE study have been published elsewhere^38–40^. Collection of data and biological samples complied with standardized procedures in all participating centres and was conducted in accordance with the principles of the Declaration of Helsinki. Cases of sPTB were defined as those women who delivered before 37 weeks’ gestation, and controls as those who delivered at or after 37 weeks’ gestation.

- Cork, Ireland.
  The subset of SCOPE participants included in this study from the Cork, Ireland centre consisted of 55 cases of sPTB and 102 controls, matched to cases according to maternal age (±3 years) and maternal body mass index (BMI; ±5 kg/m^2^)*.
- Auckland, New Zealand.

The subset of SCOPE participants included in this study from the Auckland, New Zealand centre consisted of 55 cases of sPTB and 109 controls, comprising 56 controls matched to cases according to maternal age (±3 years), and 53 controls matched to cases according to both maternal age (±3 years) and BMI (±5 kg/m^2^)*.

(*Our intention was to match each case to two controls, however, in Cork eight control subjects were excluded due to misclassification or lack of data; in Auckland one maternal age and BMI control was reclassified to a maternal age control and another was excluded due to technical difficulties).

### Data collection

Demographic information including maternal age and BMI was collected at the time of recruitment (15 ± 1 weeks’ gestation). History of vaginal bleeding and any infections during the pregnancy were also recorded via interview at the initial visit. The infant characteristics were recorded at birth and included birth weight, customised birth weight centile, and whether the infant was small, normal, or large for gestational age.

### Outcome

Spontaneous preterm birth was defined as any birth that occurred before 37 weeks due to spontaneous onset of labour or premature rupture of membranes. Gestational age (GA) was estimated by last menstrual period (LMP) and confirmed by an ultrasound dating before 16 weeks of gestation. A discordance of seven or more days between LMP and ultrasound dating or an unsure LMP led to the estimation of GA exclusively by early ultrasound parameters. Term birth for the control group was determined as delivery after 37 weeks of gestation. Early sPTB before 34 weeks was considered for subgroup analysis.

### Sample collection and storage

Maternal blood samples were collected in two 6 mL vacutainers by venepuncture at 15 and 20 weeks (±1 week). Following clot formation, the vacutainers were centrifuged at 3,000 rpm for 10 min at 4 °C, followed by a second centrifugation at 4,000 rpm for 10 min at 4 °C. After centrifugation, the resulting serum (supernatant) was pipetted into a sterile tube and aliquots of 250 µL were dispensed into cryotubes. All samples were stored at −80 °C. The study followed standard best practice procedures for repositories in all steps of sample collection and storage, registering all SPREC (Standard PREanalytical Coding) data accordingly in an online database^42^.

For this study, two 250 µL maternal serum samples were obtained from the biobank for each participant, one from each time point; 15- and 20-weeks’ gestation (±1 week).

### Sample preparation and extraction

The serum samples underwent extraction and derivatization procedures based on the 2010 protocol of Smart *et al*.^43^. Briefly, samples were thawed on ice at 4 °C and transferred from cryotubes to 1.5 mL microcentrifuge tubes. An internal standard (IS; 20 µL of 10 mM L-Alanine-2,3,3,4-d4) was added to all samples and the sample-IS mix was vortexed for 1 min. Samples were dried for 4 h at 0.8 HPa in a centrifugal vacuum concentrator with a −104 °C refrigerated vapour trap (Thermo Fisher Scientific Savant SC250EXP SpeedVac Concentrator with Savant SP5121P Refrigerated Vapour trap). Metabolites were extracted using 50% and 80% cold methanol-water solution (−20 °C, v/v). Specifically, 500 µL of 50% cold methanol-water solution was added to all samples, followed by vortexing for 1 min and centrifugation at 3,000 rpm for 5 min at −4 °C. After centrifugation, the supernatant was transferred to a fresh chilled microcentrifuge tube (kept on dry ice). Then, 500 µL of 80% methanol-water solution was added to the pellet and this was centrifuged at 3,000 rpm for 5 min at −4 °C. The supernatants obtained from both extraction steps were combined and dried in the centrifugal vacuum concentrator for 4 hours at 0.8 HPa, with a −104 °C refrigerated vapour trap. Dried extracted samples were stored at −80 °C prior to derivatisation.

Negative controls were produced by subjecting an empty microcentrifuge tube to the same processing as the samples. Pooled quality control samples (QC) were produced by pooling a small amount from every sample, mixing, and then making aliquots of the same volume as the samples.

Derivatization was carried out using methyl chloroformate (MCF). Samples were derivatised in batches of 18–24, on the same day that they were analysed on the GC-MS. Samples were re-suspended with 400 µL of 1 M sodium hydroxide and were transferred to silanised glass tubes, followed by addition of 334 µL of methanol and 68 µL of pyridine. The sample was placed on a vortexer for the remainder of the derivatization process at ~1,500 rpm. The rate limiting step began from the addition of 40 µL of MCF. A second addition of 40 µL of MCF was made, 30 sec later. After another 30 sec, 400 µL of chloroform was added to extract the alkylated derivatives from the reaction mixture. After 10 sec, 400 µL of sodium bicarbonate (50 mM) was added. Centrifugation was used to separate the aqueous layer from the chloroform layer. After centrifugation, the aqueous layer was removed and the remaining chloroform extract was dehydrated by the addition of sodium sulphate (~0.3 g). The remaining liquid was then transferred to an amber glass GC-MS vial with a glass 33 µL insert.

### Gas chromatography – mass spectrometry (GC-MS) analysis

The GC-MS instrument parameters were based on Smart *et al*.^43^, with modifications. One microliter of sample was injected for analysis. The injector was set to 290 °C in splitless mode. The column flow was maintained at 1.0 mL min-1 in constant flow mode. The column was a fused silica ZB-1701 30 m long, 0.25 mm inside diameters, with a 0.15 µm stationary phase constituting of 86% dimethylpolysiloxane and 14% cyanopropylphenyl (Phenomenex). Instrument grade helium (>99.99%, BOC) was used as the carrier gas for the analysis. The detector was run in positive-ion, electron-impact ionisation mode, at 70 eV electron energy. Identification of compounds was carried out using mass spectra acquired in scan mode from 38 to 550 atomic mass units. The Cork samples were analysed on an Agilent 7890 A gas chromatograph coupled to an 5975 C inert mass spectrometer. The Auckland samples were analysed on a Thermo Scientific Trace GC Ultra gas chromatograph coupled to an ISQ mass spectrometer.

### Data extraction and compound identification

Data processing was semi-automated. The raw files obtained from the GC-MS were converted into common data form (cdf) format for analysis and were deconvoluted and identified using the Automated Mass Spectral Deconvolution and Identification System (AMDIS - http://www.amdis.net/)^44^ from an in-house mass spectral library for MCF-derivatised metabolites (~210 compounds) developed by Silas Villas-Boas. The library contained mass spectra predominantly from certified reference standards. In addition to the in-house library, the National Institute of Standards and Technology (NIST) mass spectral library (NIST14, 163,198 compounds) was also used to identify peaks in the raw chromatograms. Since AMDIS is not able to batch deconvolute with the entire NIST library, a NIST subset library was constructed employing a method developed by Elizabeth McKenzie using pooled quality controls.

The NIST subset was produced using the top five results for each feature from the Agilent Chemstation PBM (Probability Based Matching) deconvolution program. MassOmics in house software (version 2.3) was used to create the subset library. MassOmics is an R script based on the XCMS R package^45^, with a Windows graphic user interface (GUI) developed by Ting-Li Han. MassOmics used XCMS and the AMDIS report to integrate the peak areas for each of the identified metabolites. The summary report obtained from running the MassOmics script was then checked, and peaks with low ID hits, or with large retention time shift, and laboratory contaminants were removed.

Data was checked against negative controls to identify and remove background contaminants. Peaks that were not extracted correctly with XCMS were integrated separately using the Ion Extractor feature in MassOmics. Co-eluting peaks underwent manual integration. The relative abundance obtained for each metabolite was normalised to the internal standard (Alanine-d4). After internal standard normalisation, the remaining technical variation was corrected for by analytical batch median centering using the samples. An analytical batch was defined as ~25 injections, comprising ~18 samples, 4 QC’s, one Alkane series, one negative control, and one standard mixture.

### Statistical analysis

Statistical analysis of the normalised mass spectral data was performed using R 3.4.3 (https://www.R-project.org)^46^. Data was analysed separately for each site. Clinical predictors were analysed for univariate associations with preterm birth, and a multivariate logistic regression model predicting preterm birth with these predictors was selected using stepwise logistic regression with AIC, starting from an intercept only model, followed by backward elimination of variables with multivariate p > 0.05. Mann-Whitney tests were used to analyse the difference in metabolite levels between cases and controls. Ratios between the 20 and 15-week levels of each metabolite were also assessed in this way, to identify situations where changes from baseline rather than absolute metabolite levels were associated with case status. To adjust for multiple comparison testing, false discovery rates (FDR) were calculated for each comparison using the Benjamini-Hochberg procedure^47^. Metabolites with an estimated FDR < 0.05 are reported.

Where potential metabolite predictors were identified, we then built logistic regression and random forest models using the selected clinical factors and metabolites. For logistic regression, we assessed whether one or more metabolites improved the area under the receiver operator characteristic curve compared to the model based on clinical predictors alone^48,49^. The average case-control difference in the linear predictor taken from the selected multivariate logistic regression model was also compared to average linear predictor differences from permuted data, to form a permutation test of model utility. Specifically, the model was refit to data where the response category was permuted to destroy any true association with the predictors. One thousand permutations were performed; the model was considered significantly better than random if the average linear predictor difference for the real data was better than 95% of the permuted replicates (p < 0.05). The random forest was assessed via a permutation test based on the out-of-bag error rate.

To assess whether expanding the set of predictors might improve accuracy, the sparse partial least squares discriminant analysis (PLS-DA) method from the mixOmics package was employed^50^. The clinical variables, log transformed 15 and 20-week metabolite intensities, and 20 to 15-week ratios were used as candidate predictors. Up to three components with five predictors each were used. The number of components was selected based on the Mahalanobis distance balanced error rate using 10-fold cross validation, averaged over 10 repeats. This error rate was also used to assess the significance of this model via a permutation test.

The above procedures were repeated for comparing preterm birth <34 weeks to the entire control group.

## Results

### Participants

Participant demographic and outcome characteristics are shown in Table 1. These show the success of the matching strategy: similar BMI and age in case and control groups. T-tests comparing case and control were insignificant (Cork p-values 0.68 for age, 0.47 for BMI; Auckland 0.66 and 0.54 respectively). The expected association between birthweight and gestational age category is also observed. Potential clinical predictors are shown in Table 2. For Auckland data, smoking was the only significant clinical predictor in univariate models, and the multivariate model selection also chose a model with smoking only (odds ratio = 4.9; p-value = 0.03). When considering preterm birth <34 weeks, smoking was no longer a significant predictor comparing preterm cases <34 weeks to the entire control group. For the Cork data, having vaginal bleeding before 15 weeks’ was the only significant univariate predictor of sPTB (<37weeks); multivariate model selection again chose a model with this variable only (odds ratio = 2.8; p-value = 0.006). The selected multivariate model for preterm birth <34 weeks included vaginal bleeding and being a current smoker, and history of miscarriage. However, the permutation test indicated overfitting (p = 0.27) both of this model and the model including only current smoker and vaginal bleeding (p = 0.19); accordingly, this model was reduced to include vaginal bleeding only, which was significantly better than random (p = 0.01).

**Table 1:** Medians (Lower and Upper Quartiles) or Counts (%) for Matching and Outcome Variables.

	Cork		Auckland	
	Term Birth N = 109	Spontaneous Preterm Birth N = 55	Term Birth N = 102	Spontaneous Preterm Birth N = 55
Maternal BMI*	23.4 (21.8, 25.8)	24.0 (21.6, 26.3)	23.5 (21.7, 26.0)	23.5 (21.5, 27.0)
Maternal Age (years)*	30 (27, 32)	30 (27, 34)	31 (28, 33)	32 (29, 34)
Birthweight (g)	3600 (3350, 3830)	2520 (2045, 2795)	3560 (3320, 3884)	2540 (2173, 2995)
Gestational age at delivery (weeks)	40.6 (39.9, 41.3)	35.3 (33.7, 36.4)	40.4 (39.3, 41.1)	35.7 (34, 36.4)
Gestational age at delivery				
<28 weeks	N/A	3 (6%)	N/A	3 (6%)
28–32 weeks		4 (7%)		2 (4%)
32–34 weeks		9 (16%)		8 (15%)
34–37 weeks		39 (71%)		42 (76%)
Preterm Premature Rupture of Membranes				
Yes	N/A	29 (53%)	N/A	25 (45%)
No		26 (47%)		30 (55%)

*t-tests: Cork p-values 0.68 for age, 0.47 for BMI; Auckland 0.66 and 0.54 respectively.

**Table 2:** Medians (Lower and Upper Quartiles) or Counts (%) for potential clinical predictors for sPTB <37 weeks and <34weeks.

	Cork					Auckland				
	Term Birth N = 109	Spontaneous Preterm Birth <37w N = 55	p-value <37w vs term	Spontaneous Preterm Birth <34w N = 16	p-value <34w vs term	Term Birth N=102	Spontaneous Preterm Birth <37w N = 55	p-value <37w vs term	Spontaneous Preterm Birth <34w N = 13	p-value <34w vs term
Maternal height (cm)	165 (161, 168)	165 (160, 168)	0.99	167 (160, 169)	0.48	165 (161, 169)	165 (162, 168)	0.8	164 (162, 168)	0.53
**Infant sex**										
Female	47 (43%)	24 (44%)	>0.99	7 (44%)	1 *	47 (46%)	24 (44%)	0.9	4 (31%)	0.38*
Male	62 (57%)	31 (56%)		9 (56%)		55 (54%)	31 (56%)		9 (69%)	
**Smoking in pregnancy**										
no smoking	82 (75%)	40 (72%)	0.78 §	10 (62%)	0.33*	92 (90%)	44 (80%)	0.06* § 0.03*,^†^	12 (92%)	1*^,§^
quit	17 (16%)	8 (15%)	0.66^†^	3 (19%)		7 (7%)	4 (7%)		1 (8%)	
current smoker	10 (9%)	7 (13%)		3 (19%)		3 (3%)	7 (13%)		0	
Fertility Treatment	7 (6%)	2 (4%)	0.72*	2 (13%)	0.32*	11 (11%)	7 (13%)	0.92	2 (15%)	0.64*
Previous Miscarriage	15 (14%)	10 (18%)	0.61	4 (25%)	0.26*	15 (15%)	6 (11%)	0.67	2 (15%)	1*
**Gravidity**										
1	91 (83%)	43 (78%)	0.55*	12 (75%)	0.39*	73 (72%)	37 (67%)	0.36*	8 (62%)	0.35*
2	13 (12%)	10 (18%)		2 (13%)		23 (23%)	11 (20%)		3 (23%)	
3 or more	5 (5%)	2 (4%)		2 (13%)		6 (6%)	7 (13%)		2 (15%)	
Vaginal bleeding before 15 w visit	23 (21%)	23 (42%)	0.01	9 (56%)	0.01*	22 (22%)	14 (25%)	0.72	3 (23%)	1*
Any infection before 15 w visit^a^	26 (24%)	11 (20%)	0.72	5 (31%)	0.54*	45 (44%)	24 (44%)	>0.99	6 (46%)	1*

P-values are from tests of case-control differences using the Mann-Whitney test (Continuous variables), Chi-squared test (categorical variables with adequate counts) or Fisher’s exact test (*). ^§^p-value for the three categories. ^†^p-value for current smoker vs no smoking in pregnancy. ^a^Any infection during pregnancy means upper respiratory or urinary tract infection, pyelonephritis, gastrointestinal infection, vaginal candidiasis or other infections.

### Metabolites identified

In the Cork samples, 176 compounds were detected. Of these, 77 were identified using an in-house library of reference standards and the remaining compounds were identified using mass spectrum alone (NIST 2014 mass spectral library). Of the in-house library matches, 61 were putatively identified (80–100% match to a reference standard) and 15 were tentatively identified (60-79% mass spectral match). Of the NIST14 library identifications, 25 were putatively identified (80–100% mass spectral match), 58 were tentatively identified (60–79% mass spectral match), and three were unknown (<60% mass spectral match).

In the Auckland samples, 142 compounds were detected. Of these, 50 were identified using an in-house library of reference standards and the remaining compounds were identified using mass spectrum alone (NIST 2014). Of the in-house library matches, 41 were identified, eight were tentatively identified, and one was unknown. Of the NIST14 library identifications, 51 were putatively identified, 34 were tentatively identified, and seven were unknown.

### Metabolites and performance of predictive models

**Figure 1:**
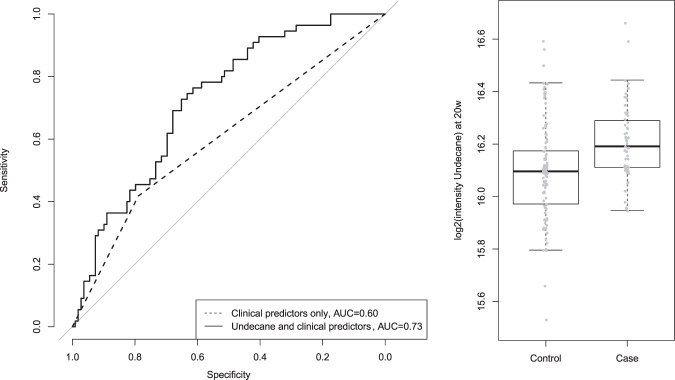
Prediction model for sPTB <37 weeks: Comparison of ROC curves using clinical predictors only (vaginal bleeding) and clinical predictors with log undecane intensity for the Cork cohort; with comparison of log_2_ intensity for undecane across case (Preterm Birth < 37w) and control (Term birth) individuals. Adapted from Souza RT^63^.

Table 3 shows the metabolites associated with preterm birth <37 weeks’ and preterm birth <34 weeks’, at each location and at each gestational time point (15 weeks or 20 weeks); ratios between the 15 and 20 week values were also assessed. Three metabolites detected at 20 weeks of gestation in the Cork subset were found to be significantly associated with sPTB (FDR < 0.05) when compared to term births: undecane, dodecane and decane. All three were found to have higher abundance in sPTB cases. Adding the natural log intensity of undecane to the clinical predictors in a logistic regression model estimates an odds ratio of 1.9 for a 1 standard deviation increase in log (undecane). This is significantly different from 1 (p = 0.0006), and the model as a whole was significant via permutation test (p = 0.001). The other metabolites were correlated with undecane (Pearson’s correlation for log intensities r = 0.87 decane, r = 0.89 dodecane) (Suppl. Fig. a). Consequently, results were similar for the other metabolites added individually to the model but adding multiple metabolites did not improve the model further. Figure 1 shows the receiver operating characteristic (ROC) curves for the clinical model only (area under the curve 0.60), and the clinical model with the addition of undecane (area under the curve 0.73) predicting sPTB <37 weeks. Supplementary Fig. b shows the ROC curve for the models predicting sPTB <34 weeks (Suppl. Fig. b).

**Table 3:** Metabolites significantly associated with sPTB <37 weeks and <34 weeks.

SCOPE site	Gestational age at sample collection	Metabolite	CAS number	P-Value*	FDR	Direction of association and PTB category
Auckland	15 weeks	None	—	—	—	—
Auckland	20 weeks	None	—	—	—	—
Auckland	Ratio between 15 and 20 weeks’ gestation	None	—	—	—	—
Cork	15 weeks	None	—	—	—	—
Cork	20 weeks	Undecane	1120-21-4	3.73E-05	0.007	Higher in sPTB <37weeks
		Dodecane	112-40-3	8.11E-05	0.007	Higher in sPTB <37weeks
		Decane	124-18-5	7.06E-04	0.04	Higher in sPTB <37weeks
Cork	Ratio between 15 and 20 weeks’ gestation	None	—	—	—	—

^*^Mann Whitney U test.

Similarly, the random forest model using log(undecane) and vaginal bleeding was significantly better than random (p = 0.002) although the classification success was modest (70%). However, models also including log(decane) or log(dodecane), or both, had error rates similar to those based on permuted data (p = 0.29, 0.08 and 0.08, respectively).

Sparse PLS-DA also produced a 1-component model for the Cork preterm birth data with an error rate that was significantly better than error rates produced for random permutations (p = 0.01). This model again included undecane, dodecane, decane, and vaginal bleeding. A fifth predictor, stearic acid measured at 15 weeks’ gestation, was present; however, its loading was small, and it did not have an odds ratio significantly different from 1 (p = 0.07) when incorporated into the logistic regression with vaginal bleeding and undecane.

No metabolites or 20–15 week ratios met the false discovery rate threshold for the Cork <34 weeks data, for either preterm <37 weeks or <34 weeks in the Auckland data, nor were the sparse PLS-DA models significant (p = 0.06 preterm birth <34 weeks Cork; p = 0.07, preterm birth <37 weeks Auckland; p = 0.48 preterm birth <34 weeks Auckland).

Log transformed Undecane did not meet the FDR threshold for association with preterm birth <34 weeks in the Cork data (compared to birth ≥ 37 weeks), but was associated with preterm birth <37 weeks. We therefore examined the sensitivity of our model to the threshold for preterm birth. The odds ratio associated with a 1 sd change in log(Undecane), in a model also including vaginal bleeding, when the divider between preterm birth and term birth was reduced to 36, 35 and 34 weeks were 2.1 (p = 0.0009), 2.2 (p = 0.0014), and 1.7 (p = 0.0571) respectively.

## Discussion

We have analysed potential predictors of sPTB using serum samples and clinical data from Cork and Auckland participants of the SCOPE study, an international cohort of low-risk nulliparous women. An untargeted metabolomics approach was applied to serum samples collected at 15 and 20 weeks of gestation. More than one hundred metabolites were identified in each subset (Cork and Auckland). As expected for the metabolomics method employed, the most common classes of metabolites were fatty acids, followed by amino acids. Only three metabolites from the 20-week serum of Cork participants were found to be significantly associated with sPTB. Vaginal bleeding before 15 weeks, and smoking during pregnancy were the only clinical factors associated with sPTB, in Cork and Auckland subsets, respectively. In the Cork cohort, adding undecane to a multivariate logistic regression model for predicting sPTB improved its performance over a model with vaginal bleeding alone. This improvement is robust to the definition of preterm birth, persisting when the preterm threshold was decreased to 36 or 35 weeks; at 34 weeks the improvement is no longer significant. We note that, as depicted in Fig. 1, there are clear average differences in undecane levels between case and control groups, but also substantial overlap, limiting the utility of the metabolite measurements in clinical practice.

There is a biologically plausible explanation for observing elevated alkanes (decane, undecane, dodecane) in the serum of mothers who had sPTB. Oxidative stress may lead to degradation of cell membranes by lipid peroxidation, followed by conversion of polyunsaturated fatty acids to volatile alkanes. The association of several oxidative stress-associated processes and sPTB have been previously reported^51–53^, and oxidative stress has been associated with elevated alkane levels in gastroenteric disease, lung disease, and other chronic diseases of metabolism^54–56^. Glutathione, an important intracellular antioxidant, has been found to be decreased in maternal and umbilical cord blood of very low preterm neonates and their mothers^57^. Preterm birth seems to be associated with depletion of glutathione, reinforcing a possible increased oxidative status and lower antioxidant capacity. However, failure to demonstrate elevated alkanes among sPTB cases in the Auckland cohort limits our confidence, and there was no evidence these alkanes were associated with early sPTB (<34 weeks) at either study site. In addition, hypotheses describing the role of reactive oxygen species generation, metabolic and inflammatory imbalance and many other downstream mechanisms (telomerase reduction, cell apoptosis and senescence, etc.) caused by oxidative stress activation^51^ do not clarify whether those mechanisms are trigger factors or consequences of underlying conditions/alterations resulting in preterm PROM and/or spontaneous onset of preterm labour.

The differences across study sites in the associations between sPTB and clinical factors such as smoking and vaginal bleeding in this case-control analysis also suggest that there are differences in the Cork and Auckland populations. The alkanes elevated in the Cork preterm birth group are present in outdoor and indoor air contaminants, which could potentially differ across study sites, providing an explanation for this observation. Association of environmental exposures with maternal and perinatal health have been reported for many years but are not yet well established^58,59^. Further investigation of individual pollutant exposure would be necessary to confirm whether elevated alkanes are associated with environmental exposure. It is also possible that technical rather than biological variability accounts for some of the differences observed across sites. Samples from the different sites, while analysed using the same protocols, were run on different platforms by different technical personnel. Thirty-four fewer metabolite species were detected in the Auckland samples, suggesting reduced sensitivity. In particular, two of the three metabolites elevated in cases in Cork (decane and dodecane) were not identified in the Auckland samples.

We also examined prediction of very preterm birth (<34 weeks). No clinical predictors were significant in the Auckland cohort, but vaginal bleeding before 15 weeks was associated with very preterm birth, as well as preterm birth, in the Cork cohort.

To the best of our knowledge, there are few studies in the literature applying metabolomics techniques to understand sPTB in asymptomatic pregnant women^33,60–62^. Previous studies have used between 20 and 70 samples of a variety of biofluids (amniotic and cervicovaginal fluid, as well as serum), equally divided between cases and controls. There is also a diversity of pregnancy stages and analytical techniques, so it is not surprising that there is little overlap in the specific compounds identified. However, Virgiliou *et al*. also suggested that the changes in amino acids and lipids they observed could be related to oxidative stress^62^.

Our study has strengths and limitations. Cases and controls were selected from a large cohort comprised of low-risk nulliparous women enrolled in early pregnancy and containing a high standard biobank. Several procedures were employed to assure data, sample, and analysis compliance and reliability according to Standard Operating Protocols. Shortcomings of our study include lack of data regarding cervical length, a previously reported risk factor, and assaying of the metabolome of the two cohorts at different times on different equipment. Genetic factors may also be contributors to PTB risk however these were not assessed in this study. In addition, we have not investigated predictive metabolites for the different preterm birth subtypes (spontaneous onset of preterm labour or preterm premature rupture of membranes), focusing instead on an early sPTB (<34 weeks) subgroup, due to the increased morbidity in this specific group. Using data and samples from women of different populations (Cork, Ireland; Auckland, New Zealand), enabled us to compare reproducibility of our technique and also to discuss possible local drivers for sPTB (Alkane pollutants in Cork, Ireland, for instance). Cork and Auckland samples were analysed by different laboratory experts and instruments and as such, the reproducibility and sensitivity differed across the sites.

While our finding of an association between elevated alkanes and sPTB at the Cork site is preliminary, it raises several interesting questions to pursue in the future. What differences are typical or expected across geographically distant study sites? What role might exposure to exogenous pollution sources, including alkanes, play in preterm birth? And finally, how might oxidative stress trigger, or be triggered by, processes leading to spontaneous preterm birth?

## Supplementary information

Supplementary Figures

## Data Availability

The analysis in this study used the information from two centres from the SCOPE Consortium that has the ownership of data. Any requirement regarding this data can be directed to Prof. Philip N Baker.
