# Supplementary material for: Trace biomarkers associated with spontaneous preterm birth from the maternal serum metabolome of asymptomatic nulliparous women – parallel case-control studies from the SCOPE cohort
Source: Sci Rep. 2019 Sep 23;9:13701. doi: 10.1038/s41598-019-50252-7 (PMC6757051; doi:10.1038/s41598-019-50252-7)
Supplement: Supplementary file 1 — Supplementary Figures [file 41598_2019_50252_MOESM1_ESM.pdf]

RESEARCH ARTICLE

Trace biomarkers associated with spontaneous preterm birth from the maternal serum metabolome of asymptomatic nulliparous women – parallel case-control studies from the SCOPE cohort

Authors

Renato T. Souza, Elizabeth J. McKenzie, Beatrix Jones, Jamie V. de Seymour, Melinda M. Thomas, Erica Zarate, Ting Li Han, Lesley McCowan, Karolina Sulek, Silas Villas-Boas, Louise C. Kenny, José G. Cecatti, Philip N. Baker

a)

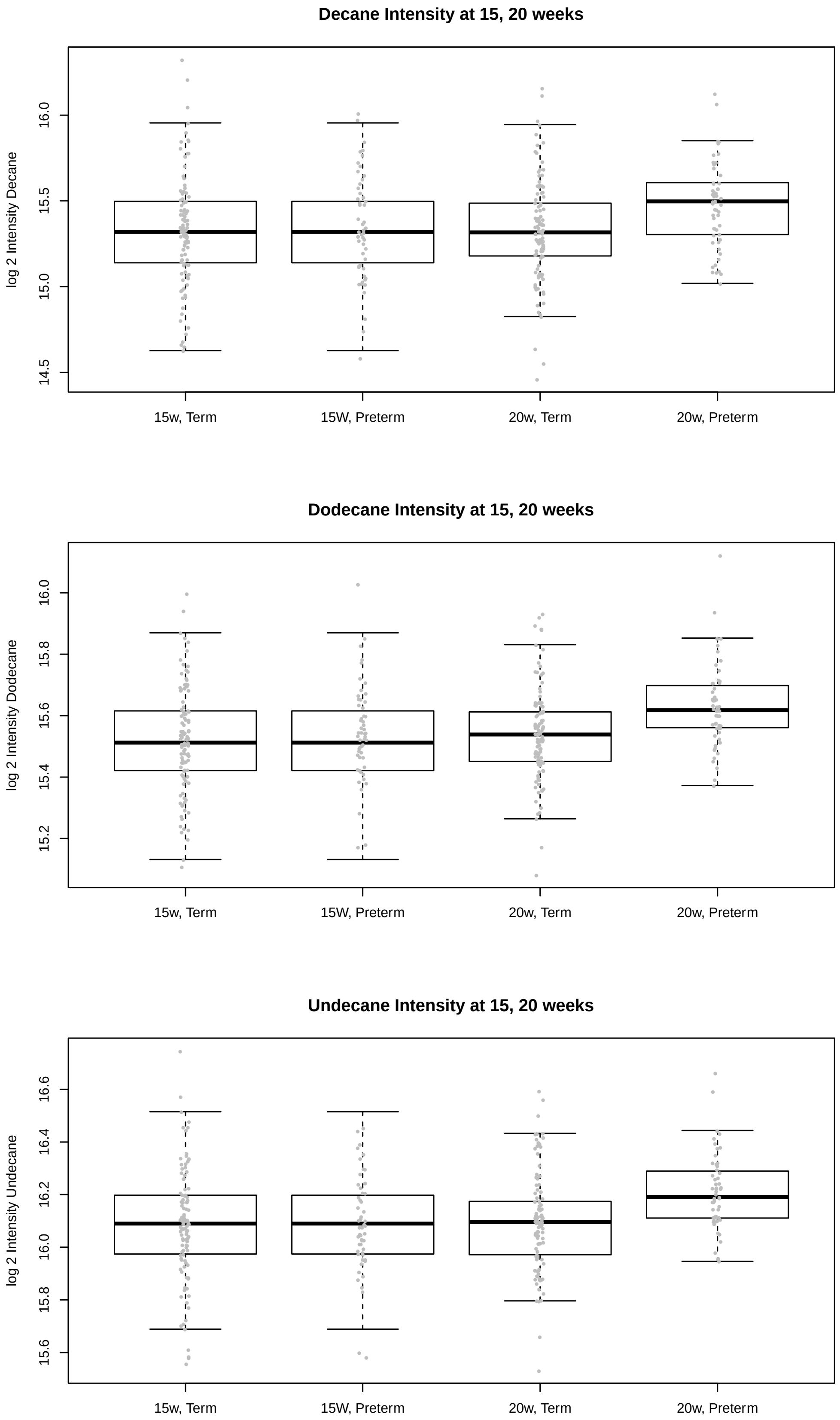
Decane Intensity at 15, 20 weeks
log 2 Intensity Decane
14.5
15.0
15.5
16.0
15w, Term
15W, Preterm
20w, Term
20w, Preterm
Dodecane Intensity at 15, 20 weeks
log 2 Intensity Dodecane
15.2
15.4
15.6
15.8
16.0
15w, Term
15W, Preterm
20w, Term
20w, Preterm
Undecane Intensity at 15, 20 weeks
log 2 Intensity Undecane
15.6
15.8
16.0
16.2
16.4
16.6
15w, Term
15W, Preterm
20w, Term
20w, Preterm

b)

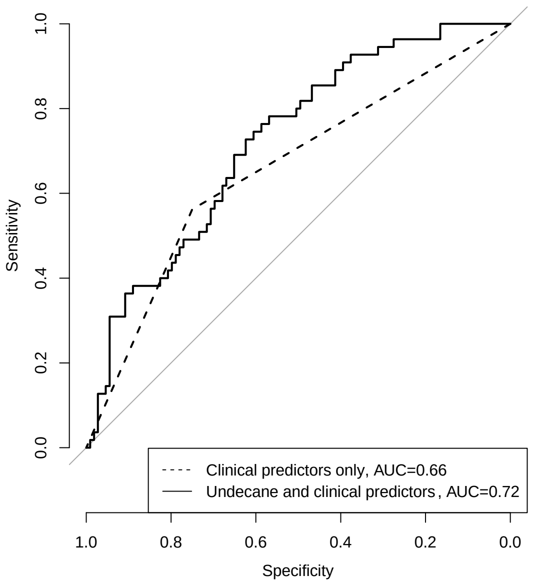
1.0
0.8
0.6
0.4
0.2
0.0
Sensitivity
Clinical predictors only, AUC=0.66
Undecane and clinical predictors , AUC=0.72
1.0
0.8
0.6
0.4
0.2
0.0
Specificity
